# The necessity of gut microbiome characterization in diseases prevention and therapy

**Published:** 2017

**Authors:** Sama Rezasoltani, Ehsan Nazemalhosseini Mojarrad, Mohsen Norouzinia, Hamid Asadzadeh Aghdaei

**Affiliations:** 1 *Basic and Molecular Epidemiology of Gastrointestinal Disorders Research Center, Research Institute for Gastroenterology and Liver Diseases, Shahid Beheshti University of Medical Sciences, Tehran, Iran *; 2 *Gastroenterology and Liver Diseases Research Center, Research Institute for Gastroenterology and Liver Diseases, Shahid Beheshti University of Medical Sciences, Tehran, Iran*

**To The Editor**

 Most cells in human body are microbial (almost 90%) and near a million microbial genes are expressed within us compared to only about 20500 human genes. Each person’s profile of gut microbiome is constantly influenced by different factors including, but not limited to, sex, age, genetics, diet, lifestyle and environment. Although each individual’s microbial profile is unique, the relative abundance and distribution of microbial species is similar among the healthy, resulting in the preservation of one’s overall health (1). There is a strongly mutualistic relationship between gut microbiota and human. Thereby, dysbiosis of healthy gut microbiota results in deterioration of this mutualistic relationship and causes many diseases like obesity, metabolic syndrome, type 1 diabetes, type 2 diabetes, inflammatory bowel disease, irritable bowel syndrome, colorectal cancer and celiac disease (2,3).

It is critical to note that gut microbiota composition is different and distinct in people from developed and developing countries and may be responsible for the conundrum of reduced vaccine efficacy in developing countries. The specific characteristics of the gut microbiota are likely associated with cultural habits, host diet, and socioeconomic status (3). Recently, researchers debate that geography is the most powerful predictor of the gut microbiota compound. Differentiation in gut microbiota composition has been depicted in African, United States, European and Amerindian populations (4). It is demonstrated that gut flora composition is different between urban and rural populations; for example, communities from multiple rural zones in Russia showed similarities within each zone and are dominated by novel community (*Firmicutes* and *Actinobacteria*) that is related to the healthy gut. Long-term diet was discovered to be one of the important factors linked to gut microbiota composition. High consumption of starch-rich potatoes and bread, that are main staple foods and natural products in rural Russian communities which are accessible even to low-income socioeconomic individuals, creates this distinction in gut microbiota composition. Meanwhile, lack of this special gut microbiota component in Western cohorts is linked with low consumption of resistant starch and growth of food industrialization in the West vs. developing countries .The resemblance between the gut microbiota of Russian city populations and inhabitants of Western cities are maybe associated with Western lifestyle, higher social standards and food habits, which is especially reflected in the diet in the form of processed food and high consumption of meat products (5).

It is amazing to declare that the effect of aging on the gut microbiota composition of Europeans was country-specific. Marked country-age interactions were identified for Italian and German study populations. These interactions were inverse for the predominant bacterial groups *Bacteroides*-*Prevotella* and *Eubacterium* *rectale/ Clostridium* *coccoides*. Higher proportions of *Enterobacteria* were observed in all elderly volunteers independent of the area (4). 

The NIH Common Fund Human Microbiome Project (HMP) was founded and run in 2008, with the aim of vast characterization of human gut microbiome and analyzing its role in human health and disease. HMP has been established in the USA, Europe, Canada, Australia, Japan, Korea, and China (6).

Asia and the Middle East differ markedly in its regions which are populated by various ethnic groups that maintain their own dietary habits and cultures. In one study, the composition of the Japanese gut microbiome was shown more abundant in the phylum *Actinobacteria*, especially in the genus *Bifidobacterium*, compared to other nations (7).

Another study addressed the gut microbiota composition in five countries spanning temperate and tropical areas of Asia. The majority in Japan, China, and Taiwan harbored *Bifidobacterium/Bacteroides* type, whereas those from Indonesia and Thailand greatly harbored *Prevotella* type (8). Now, it is necessary to establish these types of projects in more nations from the continent of Asia more seriously. Considering the importance of these types of projects, it is time for getting the latest knowledge of the filed including: transcripteomic, metabolomic, proteomic and metagenomic structure of the gut. Moreover, interventional procedures are necessary to manipulate microbiota composition by probiotics and/or prebiotics and fecal transplantation as a realistic therapeutic strategy for several infectious, inflammatory and other disorders; these initiative require facilities such as appropriate high-throughput sequencing facility and appropriate protocol for analysis. Advances in independent investigations in developing countries, experimental studies and bioinformatics panels have improved our knowledge, which can be then applied clinically to improve therapeutic approaches and outcomes.

## Conflict of interest

 The authors declare that they have no conflict of interest.
